# Delirious mania in a 19 year-old Ukrainian refugee

**DOI:** 10.1192/j.eurpsy.2025.1232

**Published:** 2025-08-26

**Authors:** N. Goh, I. Onwuemelie, S. Patel

**Affiliations:** 1 Rehabilitation Psychiatry, National Forensic Mental Health Service; 2 Perinatal Psychiatry, Galway-Roscommon Mental Health Service; 3 Liaison Psychiatry, University Hospital Galway; 4 Liaison Psychiatry, University Hospital Portiuncula, Galway, Ireland

## Abstract

**Introduction:**

We present a case study of a 19-year old Ukrainian refugee presenting with delirious mania, with no personal or family psychiatric history. Delirious mania is a complex and severe neuropsychiatric condition that remains under-recognised in diagnostic manuals. Delirious mania describes a neuropsychiatric state where a patient is exhibits signs and symptoms of mania, delirium and often catatonia. Most of the patients described in past case reports are older in age than our patient and have a personal or family history of a mood disorder.

**Objectives:**

Our objectives were to investigate the signs and symptoms of delirious mania in this this 19 year-old patient. We sought to compare his presentation to the existing diagnoses in the Diagnostic and Statistical Manual of Mental Disorders Fifth Edition and International Classification of Diseases 11th Revision. These include delirium, mania and catatonia. In addition, we aimed to study existing literature on delirious mania as well as cross-cultural factors in formulation of this complex case.

**Methods:**

We assessed this patient daily as part of the Liaison Psychiatry team and compared his presentation to existing diagnostic criteria of delirium, mania and catatonia. We performed a literature review of delirious mania to identify suggested diagnostic criteria, management strategies and cultural considerations.

**Results:**

Our patient presented to the Emergency Department with a four-day history of bizarre behaviour and poor sleep. He had no past psychiatric history. He was admitted to the general medical ward for a medical work-up, which ultimately yielded no organic pathology. During this time, our patient fulfilled separate diagnostic criteria for delirium, manic episode and catatonia. We found that there was no overarching diagnosis within international diagnostic manuals that encompassed this patient’s presentation. However, delirious mania has been described in the literature since 1849. There are various suggested diagnostic criteria for delirious mania suggested in the literature, such as those by Bond (1980) and Fink (1999). This patient was successfully treated with intramuscular lorazepam. In our discussion, we considered various unique factors in the formulation. These included the cultural considerations given that the patient had recently entered the country as a Ukrainian refugee, difficulties in assessment and treatment due to language and cultural barriers and the limitations of psychiatric treatment on a general medical ward.

**Conclusions:**

Delirious mania continues to be unrepresented in diagnostic manuals, which in turn leads to confusion over diagnosis and treatment. Given the complexity of this neuropsychiatric condition, early recognition is key in providing effective treatment before progression into a malignant state. This would be aided by having delirious mania recognised in international classifications.

**Disclosure of Interest:**

None Declared

